# Bilateral Morganella Morganii keratitis in a patient with facial topical corticosteroid-induced rosacea-like dermatitis: a case report

**DOI:** 10.1186/s12886-017-0504-5

**Published:** 2017-06-28

**Authors:** Bei Zhang, Fei Pan, Kejian Zhu

**Affiliations:** 1 Department of Ophthalmology, Sir Run Run Shaw Hospital, Zhejiang University School of Medicine, 3 Qinchun Road East, Hangzhou 310016 Zhejiang, People’s Republic of China; 2 Department of Dermatology, Sir Run Run Shaw Hospital, Zhejiang University School of Medicine, Zhejiang, People’s Republic of China

**Keywords:** Bilateral, Keratitis, Morganella Morganii, Corticosteroid, Dermatitis

## Abstract

**Background:**

Bilateral keratitis rarely occurs in individuals without predisposing factors. Here we describe the clinical course of a patient who developed a bilateral keratitis caused by Morganella. morganii which might be associated with long term using of topical corticosteroids-containing preparations on the face.

**Case presentation:**

A 52-year-old female patient presented with marked bilateral corneal infiltration and hypopyon without any usual predisposing factors for bilateral infectious keratitis. There was diffuse erythema with itching on face before the onset of eye discomforts. Microbiological culture of materials from both corneas revealed significant growth of Morganella morganii. Topical corticosteroid-induced rosacea-like dermatitis was diagnosed by dermatologist because of the characteristic eruptions and long history of using the corticosteroids-containing cosmetic creams on her face. The corneal ulcers responded well to levofloxacin eye drops and ofloxacin ointment and healed with opacity and neovascularization.

**Conclusion:**

This case illustrates that bilateral bacterial corneal infection can develop in patients with long term using of topical corticosteroids-containing preparations on the face. To our knowledge, this is the first case of bilateral keratitis caused by Morganella morganii.

## Background

Bilateral keratitis usually occurs in predisposed individuals such as contact lens wearers, those suffering from malnutrition, immunodeficiency or patients undergoing bilateral refractive corneal surgery [1–3]. It has rarely been reported to develop in the absence of one of the predisposing factors mentioned above [4]. Morganella morganii is a Gram negative bacillus which has been reported more commonly as a pathogen for panophthalmitis and orbital infection than the cause of corneal infection [5, 6]. We report here a case of bilateral bacterial keratitis caused by Morganella morganii developed in a healthy woman with facial topical corticosteroid-induced rosacea-like dermatitis.

## Case presentation

The study was approved by the ethics committee of Sir Run Run Shaw hospital and the written informed consent for the publication of images was obtained from the patient.

**Fig. 1:**
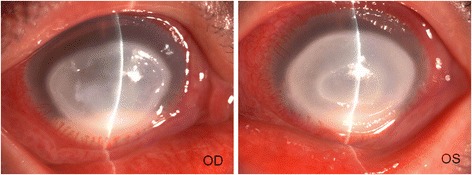
Slit lamp photograph of the right eye and left eye at first presentation, showing conjunctival injection, bilateral central large corneal ulcers with deep corneal stromal infiltration and hypopyon

**Fig. 2:**
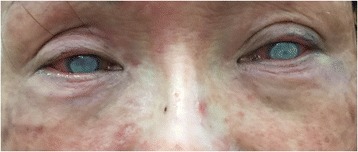
Clinical photograph at first presentation demonstrating facial papules around the eyelid and bilateral corneal ulcers

A 52-year-old female patient visited our hospital complaining of pain, redness and decreased vision in both eyes for about 4 weeks. There was no history of trauma, contact lens wear, systemic illness, pre-existing ocular disease, or ocular surgery. The patient also complained that she had the appearance of a flaming red, scaly, papule-covered face with itching before the onset of eye discomforts. She was unable to say for certain which eye had been affected firstly and she hadn’t seen any doctors before she came to our hospital. Examination revealed a vision of perception of light and remarkable conjunctival injection in both eyes. The corneal ulcers in both eyes are nearly symmetric which were about 6 × 6 mm in the central part of the cornea with marked deep stromal infiltration and hypopyon (Fig. 1). There was no evidence of chronic dacryocystitis, trichiasis, lagophthalmos, lid scarring or notching. Diffuse papules on face including eye lids were presented with pigmentation and dryness (Fig. 2). The diagnosis was infectious keratitis in both eyes, and corneal cultures of both eyes were performed. No fungal hyphae or Acanthamoeba cysts could be detected by in vivo confocal microscopy (Confoscan 3, Nidek Technologies America, Inc., Greensboro, NC, USA). The patient was started on topical 0.3% levofloxacin eye drops (Cravit; Santen, Osaka, Japan) every 30 min and 3 mg/mL ofloxacin ointment (Tarivid; Santen, Osaka, Japan) at night.

Upon taking a more detailed history, the patient revealed that she had been using corticosteroids-containing cosmetic creams on the face for about 5 months and before that she had no eruptions on her face. The use of topical corticosteroid was suggested by her beauticians and the exact type was unknown. She had history of exacerbation of the eruptions following sun exposure and rebound phenomenon on stopping the cream. The patient was referred to dermatology outpatient department, where she was diagnosed with topical corticosteroid-induced rosacea-like dermatitis. Topical tacrolimus 0.03% dermatologic ointment (Protopic; Astellas Pharma, Tokyo, Japan) was prescribed for treatment.

In view of the unusual presentation, the patient was also investigated for evidence of immunosuppression and any systemic focus of infection. Human immunodeficiency virus serology was normal. Blood counts, blood sugar levels, liver and renal function tests were all within normal limits. Physical examinations and chest X-ray also failed to reveal any evidence of systemic disease. Serum vitamin A levels were not measured but the patient did not show any evidence of vitamin A deficiency. The patient was a housewife living in urban communities with good hygienic conditions and appeared to be in good health.

**Fig. 3:**
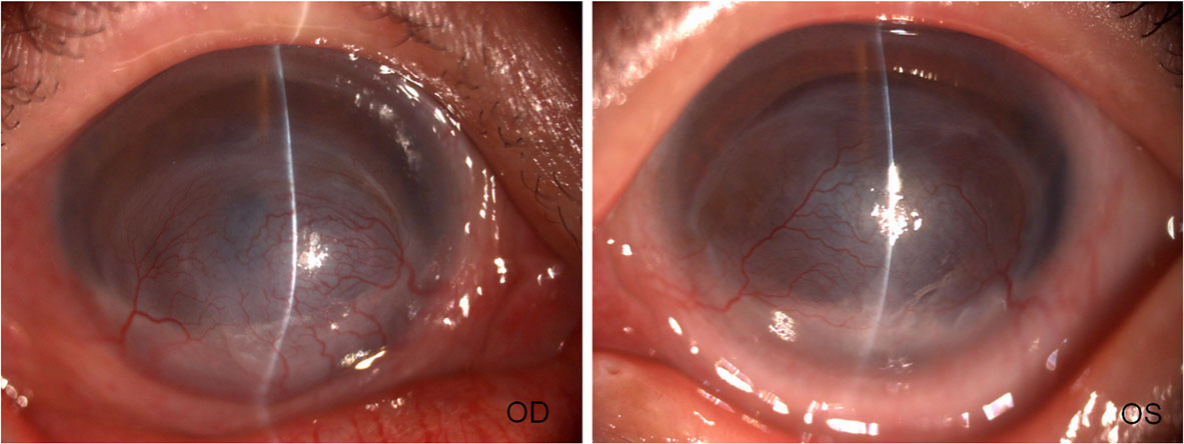
Slit lamp photograph of the right eye and left eye 2 months after the treatment demonstrating resolution of bilateral corneal ulcer with stromal opacity and neovascularization

Microbiological culture of materials from both corneas both revealed significant growth of Morganella morganii, which was sensitive to amikacin, aztreonam, ciprofloxacin, ceftriaxone, Ceftazidime, levofloxacin and tobramycin using the disk diffusion method. Topical 0.3% levofloxacin eye drops and 3 mg/mL ofloxacin ointment were administered continuously. The ulcers showed good response to medications with decrease in infiltration. Resolvement of infection with corneal scarring was noted after 6 weeks. The corneas of both eyes showed stromal opacity and neovascularization 2 months after the treatment and the vision of both eyes was hand movement before eyes (Fig. 3).

## Discussion

Bilateral infectious keratitis usually occurs in individuals with apparent risk factors, such as trauma, surgery, contact lens wearing or malnutrition [1–3]. There has only one report of bilateral keratitis caused by Pseudomonas aeruginosa developed in the absence of any obvious predisposing factor [4]. Poor hygienic conditions, lack of clean water and high susceptibility to minor eye trauma while farming were the possible factors for that patient.

In the present case, the patient had none of the usual predisposing factors for bilateral infectious keratitis. We suspected that keratitis was associated with the topical corticosteroid-induced rosacea-like dermatitis which preceded the onset of corneal infection. Corticosteroid-induced rosacea is characterized by centrofacial, perioral, and periocular monomorphic inflammatory papules distributed in areas that have been chronically exposed to topical steroids, especially of fluorinated type [7]. The skin has the appearance of rosacea-like eruptions and is rendered extremely vulnerable to bacterial, viral, and fungal infections. We presumed that long term application of corticosteroids around the eyelid could also lead to an immunocompromized state of the cornea in this patient. Minor corneal abrasions and secondary bacterial infection might be developed when she scratched the facial eruptions.

Topical corticosteroids are being misused widely on the face without a prescription from the dermatologists in China. Misuse of topical corticosteroids -containing preparations on the face and the adverse effects due to its application have been reported [8], but this is the first report of cornea infection in association with facial corticosteroids usages. We suggest that dispensing of corticosteroids must be regulated in China.

Morganella morganii is a Gram negative bacillus that belongs to the Enterobacteriaceae family and is considered as an unusual opportunistic pathogen that mainly causes post-operative wound and urinary tract infections [9]. This species is a rare cause of ocular and periocular infections, such as endophthalmitis and orbital abscess [5, 6]. Only few case of keratitis secondary to Morganella morganii has been reported [10, 11]. In our case, Facial topical steroids might induce ocular immunosuppression, thus made corneas vulnerable to be infected by opportunistic pathogens. Morganella morganii is considered as a non-negligent opportunistic pathogen because of the increased levels of resistance and virulence. This organism is a related to genera Proteus and Providencia and has a unique antibiotic sensitivity profile with natural resistance to penicillins, some cephalosporins, macrolides, lincosamides and others. On the other hand, it is sensitive to some agents including aminoglycosides, piperacillin, third and fourth generation cephalosporins, carbapenems, and quinolones [9]. In our case, the organism responded well to quinolones.

## Conclusion

This case illustrates that bilateral bacterial corneal infection could develop in patient with long term using of topical corticosteroids-containing preparations on the face. To our knowledge, this is the first case of bilateral keratitis caused by Morganella morganii.
